# Supplementary material for: Job Demand-Control-Support Model as Related to Objectively Measured Physical Activity and Sedentary Time in Working Women and Men
Source: Int J Environ Res Public Health. 2019 Sep 12;16(18):3370. doi: 10.3390/ijerph16183370 (PMC6765997; doi:10.3390/ijerph16183370)
Supplement: Supplementary file 1 [file ijerph-16-03370-s001.pdf]

Table S1. The subscales and items included in the JD-C-S model

**Sub questions and item number**	**Item text**
Demand	
1	Does your job require you to work very fast?
2	Does your job require you to work very hard?
3	Does your job require too great a work effort?
4	Do you have sufficient time for all your work tasks?
5	Do conflicting demands often occur in your work?
Control	
6	Do you have the opportunity to learn new things in your work?
7	Does your job require skills?
8	Does your job require creativity?
9	Does your job require doing the same tasks over and over again?
10	Do you have the possibility to decide for yourself *how* to carry out your work?
11	Do you have the possibility to decide for yourself *what* should be done in your work?
Support	
14	There is a quiet and pleasant atmosphere at my place of work
15	There is good collegiality at work
16	My co-workers (colleagues) are there for me (support me)
17	People at work understand that I may have a ''bad'' day
18	I get along well with my supervisors
19	I get along well with my co-workers

Each item was scored on a five-point scale from 1 to 5, corresponding to five response categories, for Demands and Control they ranged from "Very rarely" to "Very often", and for Social support from "Not true at all" to "Completely true" [9, 10].
